# The use of nutritional guidance within chiropractic patient management: a survey of 333 chiropractors from the ACORN practice-based research network

**DOI:** 10.1186/s12998-018-0175-1

**Published:** 2018-02-20

**Authors:** Mi Kyung Lee, Lyndon Amorin-Woods, Vincenzo Cascioli, Jon Adams

**Affiliations:** 1 School of Health Professions, Murdoch University, Perth, Australia; 2 Faculty of Health, University of Technology Sydney, Sydney, Australia

**Keywords:** Chiropractors, Nutrition, Nutritional guidance, Diet, Dietary supplements, Health promotion, Nutritional assessment

## Abstract

**Background:**

Food consumption and nutritional status affect an individual’s health throughout their life-course and an unhealthy diet is a major risk factor for the current global burden of chronic disease. The promotion of health and good nutrition through healthy eating requires the active involvement of all health professionals including chiropractors. This paper reports findings from the first nationally representative examination of the use of nutritional guidance within chiropractic patient management in Australia.

**Methods:**

A sample of 1000 practising chiropractors was randomly selected from the Australian Chiropractic Research Network (ACORN) practice-based research network database for a cross-sectional study and 33% participated in the online survey in November 2016. The questionnaire, based on previous designs used in similar surveys and nutrition resources developed by the National Health and Medical Research Council, was pretested prior to the survey. Pearson’s Chi square and bivariate logistic regression were undertaken to explore relationships with variables of interest.

**Results:**

The demographic details of the respondents are similar to those of the chiropractic workforce registered in Australia. Most chiropractors provided nutritional advice as part of their patient care and around a quarter provided specific dietary advice to their patients, including the use of nutrition supplements. Nutrition-related conditions most commonly encountered by the chiropractors were musculoskeletal, usually inflammatory in origin. Common nutritional assessment methods used included questioning patients to assess their nutritional and health status and physical appearance. Most of the participants provided nutritional resources to their patients in their clinics. However, the Australian Dietary Guidelines and the accompanying Australian Guide to Healthy Eating were not well utilised by the respondents. Australian chiropractors often referred patients with nutrition issues to qualified dietitians and other health professionals when deemed necessary.

**Conclusions:**

Australian chiropractors regularly provide nutritional advice and appear to acknowledge the importance of nutrition in their clinical practice especially for patients presenting with chronic disease. If chiropractors are to fulfil their potential in providing such wider public health and preventative health advice to patients, further research examining the utilisation of evidence-based nutrition resources within chiropractic patient management is recommended.

## Background

Food and nutrition are important determinants of morbidity and mortality in contemporary society [1]. Nutrition related factors (for example excess energy intake, high salt diet, high fat diet) are associated with approximately 50% of the chronic burden of disease including cardiovascular disease, hypertension, obesity and diabetes [2, 3].

The first Dietary Guidelines for Australia were developed in 1980 by the Commonwealth Health Department and the National Health and Medical Research Council (NHMRC) [4, 5] and remain in place today. The guidelines promote moderating body weight and growth, encouraging consumption of fruit and vegetables, whole grain cereals with limits on salt, sugar, fats and alcohol, promoting breastfeeding and ensuring food safety [6, 7]. Numerous recent reviews and meta-analyses have confirmed the value of these guidelines for health and longevity [8–11].

Currently the major resources for evidence-based nutrition information in Australia are the Australian Dietary Guidelines (ADG) and its companion educational food guide, the Australian Guide to Health Eating (AGHE) and the Infant Feeding Guidelines [12, 13]. The requirements for micronutrients and optimal proportion of macronutrients are outlined in the Nutrient Reference Values, a set of recommendations for nutritional intake [14]. These documents and the detailed background evidence documents are available online [12].

The ‘Go for 2 (Fruit) and 5 (Vegetable)’ campaign which promotes eating the minimum number of serves each day was based on the ADG was initiated in Western Australia and has been extended Australia-wide [15]. However, only 7% of the Australian population have been reported to meet the vegetable intake recommendations (from the Australian Dietary Guidelines) while over half (54%) met the recommendations for usual serves of fruit in the National Health Survey 2011–12 [16]. These results indicate how difficult it is to encourage most Australians to follow healthy eating practices.

The task of promoting health and good nutrition through healthy eating practices to every Australian requires the active involvement of all health professionals. It is recognised that chiropractors may provide nutrition advice as primary contact professionals and have an important role in identifying modifiable risk factors of chronic diseases as part of their patient management [17].

A systematic review of the provision of nutrition care by primary health professionals in Australia found 21 studies with 12,000 subjects [18, 19]. However, there was a lack of studies of chiropractors identified in this Australian study and also in another review of health professional nutrition education in the US [20, 21]. In contrast several surveys have been undertaken studying nutrition care in other health professions [22, 23]. The literature suggests that a majority of chiropractors within the UK, the US and Canada include nutrition as part of their patient care protocol [24, 25]. The 2015 Practice Analysis of Chiropractic conducted by the National Board of Chiropractic Examiners in the US found 97% of chiropractors were providing patients with some nutritional recommendations [26, 27]. This is an increase on previous studies in the US and Canada that indicated 80–90% of chiropractors were providing patients with nutritional advice [28, 29].

There have been no recent nationally representative studies specifically focused upon the provision of nutrition management by chiropractors in Australia. Only one small study on this topic was published with a sample of 138 chiropractors participating in 2002 [17]. In this study, 72% of the respondents were prepared to offer counselling regarding diet, while 17% were willing to provide individual health care plans. Overall, 98% of the practitioners in this previous study made dietary information available for their patients in some form.

In direct response to the important research gap in our understanding regarding the nutritional advice practices of chiropractors for patients in their care, this paper reports findings from the first large-scale study of the utilisation of nutritional guidance in Australian chiropractic practices. The study was guided by a number of specific objectives: measuring the proportion of chiropractors who apply nutritional management; identifying methods used to assess nutritional status of patients; and identifying how chiropractors guide and educate their patients regarding nutrition.

## Methods

A sample of 1000 practising chiropractors was randomly selected from the Australian Chiropractic Research Network (ACORN) practice-based research network (PBRN) database. The ACORN PBRN, founded in 2015, is independently conducted by the Australian Research Centre in Complementary and Integrative Medicine (ARCCIM) at the University of Technology Sydney [30, 31]. The ACORN database is a large, nationally representative sample across age, gender, and location of the Australian chiropractic profession containing contact details of 1680 chiropractors [32]. The analyses reported in this paper refer to a sub-study which was recruited from a randomly selected sample of 1000 practising chiropractors from the baseline ACORN PBRN database. The study reported here received approval from the Human Research Ethics Committee of Murdoch University (approval #2016/125) as well as approval from the ACORN Project Steering Committee.

Using the StatCalc routine of the Epi Info V7.15 program for cross-sectional surveys, 95% confidence limits and 80% power, a minimum sample size was calculated to be 313.

Potential participants were invited by e-mail to complete an online questionnaire in November 2016.

The questionnaire was based on instruments employed to survey chiropractors in the US, and other health professionals in Australia, Canada and New Zealand [29, 32–36] with questions modified to suit the Australian context and include commonly used nutrition resources developed by the National Health and Medical Research Council [37]. The questionnaire was then pilot tested with a focus group of practising chiropractors, researchers and academics. The final questionnaire was designed for online use [38] and comprised 22 open-ended and close-ended questions, taking approximately 15 min to answer.

The questionnaire sought information regarding: general demographic details of the chiropractor; inclusion of nutrition in their patient management; assessment of the nutritional status of their patients; methods used in practice to improve nutrition where required; resources used in providing nutritional advice; familiarity with the ADG and its eating guide; and nutrition resources provided to patients. A comments box was provided to enable participants to leave additional information. Where relevant these comments are presented in the results.

Two follow-up emails were sent to the entire random sample (*n* = 1000) and the survey remained open for a one month period. Data were downloaded from the online survey platform into an SPSS database [39]. The database was cleaned and checked and descriptive frequencies obtained. To explore relationships with variables of interest cross tabulations with Pearson’s Chi square were employed. *P* < 0.05 using a 2-tail test was regarded as significant. To explore further and adjust for demographic variables bivariate logistic regression was undertaken.

## Results

A total of 333 questionnaires were completed constituting a response rate of 33%. The demographic details of the respondents compared to the details of the chiropractic workforce as registered in Australia [40] (Table 1).

**Table 1:** Characteristics of survey respondents by gender

	Male (*n* = 201)	Female (*n* = 132)	Total	
Age (years)				
< 30	14 (41.2%)	20 (58.8%)	34 (100%)	NS
31–50	108 (54.0%)	92 (46%)	200 (100%)	NS
> 50	79 (79.8%)	20 (20.2%)	99 (100%)	*P* < 0.01
Total	201 (60.4%)	132 (39.6)	333 (100%)	P < 0.01
Educational Institution				
Macquarie University	62 (30.8%)	50 (37.6%)		NS
Murdoch University	10 (5.0%)	15 (11.3%)		NS
RMIT	78 (38.8%)	51 (38.3%)		P < 0.01
New Zealand College of Chiropractic	4 (2%)	0		
Other	47 (23.4%)	17 (12.8%)		NS
Total	201 (100%)	133 (100%)		P < 0.01
Location of Practice				
Urban	158 (78.6)	99 (74.4%)		NS
Rural	39 (19.4%)	31 (23.3%)		NS
Remote	4 (2%)	3 (2.3%)		
Total	201 (100%)	133 (100%)		NS
Type of Practice (not mutually exclusive)				
With other Chiropractor	118 (57.6%)	87 (42.4%)	205	NS
With Physiotherapist	18 (78.3%)	5 (21.7%)	23	NS
With General Practitioner	14 (77.8%)	4 (22.2%)	18	NS
Naturopath	36 (55.4%)	29 (44.6%)	65	NS
Psychologist	32 (54.2%)	27 (45.8%)	59	NS
Solo practitioner with no other health professionals	46 (61%)	29 (39%)	75	NS

When asked to estimate whether they provide nutritional advice to their patients on more than 50% of occasions, male chiropractors were less likely to do so (33%) compared to 46% for females (X^2^ 6.04, *p* = 0.014). A smaller number of practitioners (30%) stated that providing nutritional advice was not a major component of their practice (i.e. ‘never’, or to ‘less than 25 % of patients’).

Practitioners were asked about the type of health conditions presented by their patients in their practices for which they provide nutritional advice (Table 2).

**Table 2:** Health conditions encountered in chiropractic practice and nutritional advice

	Frequently/ Always (>50%) n = (%)	Sometimes n = (%)	Rarely or Never n = (%)	Total n=
Inflammatory conditions	186 (57%)	98 (29%)	45 (14%)	329
Gastrointestinal disorders	182 (55%)	85 (26%)	61 (19%)	328
Food allergies/intolerances	166 (51%)	83 (25%)	79 (24%)	328
Blood glucose (e.g. Diabetes, Hypoglycaemia)	146 (44%)	100 (30%)	83 (26%)	329
Osteoporosis	142 (43%)	116 (35%)	69 (21%)	329
Pregnancy/post-partum	121 (37%)	88 (27%)	118 (36%)	327
Weight Loss	122 (37%)	122 (37%)	84 (26%)	
Cardiovascular Disease	116 (35%)	110 (34%)	101 (31%)	327
Allergies (non-food)	116 (35%)	99 (30%)	112 (34%)	327
Anaemia	86 (26%)	97 (30%)	144 (44%)	328

The most frequently used methods of nutritional (and health) assessment by the survey respondents at the initial patient examination (‘frequently or always’ category) were questions on medications (94%), use of nutritional supplements (84%) and herbal medicines (80%), smoking (80%), use of alcohol (63%), patient’s own perception of nutritional and health status (62%), physical appearance (56%), dietary assessment (28%), anthropometric measurements including the waist/hip ratio (16%) and laboratory measures (10%).

The nutrition resources used by the participants to provide nutritional advice to patients included a range of general health books and magazines containing nutritional advice (58%), nutrition brochures/posters (46%), practice newsletters containing nutritional advice (28%) and scientific/professional journals/newsletters about nutrition (25%). No materials were provided in a minority of practices (22%). Other resources provided by the participants included ‘Facebook’ pages and online blogs, healthy recipes, specific articles e-mailed to patient as needed, and resources provided by dietitians and nutrition companies.

While most of the respondents were familiar with the Australian Dietary Guidelines (70%) only a small proportion (17%) reported regularly using the Australian Guide to Healthy Eating (AGHE) when providing nutritional guidance to patients. The use of the AGHE was more common amongst younger respondents (<30 years), but this was not a significant association in the multivariate analysis.

## Nutritional recommendations and interventions

Most of chiropractors in this study (92%) responded ‘Yes’ to the question on whether they ever recommended nutritional supplements to their patients. The most commonly recommended nutritional supplements by those chiropractors who report recommending supplements are shown in Table 3 (in descending order of ‘often’ frequency).

**Table 3:** The most commonly recommended nutritional supplements

	Often (%)	Sometimes (<50%)	Rarely (%)	Never or No response (%)
Probiotics	49	34	6	11
Essential fatty acids	47	30	9	14
Minerals	43	31	11	15
Vitamins (single or multi)	40	40	7	13
Protein	22	38	29	11
Fibre	18	39	24	19
Herbal remedies	18	31	32	19
Enzymes	13	31	35	21

Other small number of respondents (*n* = 15) made other suggestions for supplements including: homeopathic remedies, protomorphogens, glucosamine, chelating and methylating formulas, Vitamin D and B12 for vegans, curcurmin for inflammation, fats and organ meat, water, fruit and vegetable supplements.

Other nutritional guidance recommended to patients with nutritional problems (‘frequently’ or ‘always’), included: “Discussing making healthy food and drink choices (72%)”, “Suggestion of healthy recipes (40%)”, “Recommendation of nutrition brochures/books (29%)”. The ‘Australian Guide to Healthy Eating’ was recommended by a small number of participants (less than 1%). However, 8% of the respondents used ‘Eat for Health’ website that includes dietary guidelines and the Australian Guide to Healthy Eating as a resource to provide nutrition information to their patients.

Respondents also provided a diet plan (28%) and group education programs in nutrition (16%) to their patients. The list of diets recommended included FODMAP (Fermentable Oligosaccharides, Disaccharides, Monosaccharides and Polyols), Mediterranean diet, Paleo, reduced Carbohydrate diet, Ketogenic diet, low GI (Glycaemic Index) diet, CSIRO (The Commonwealth Scientific and Industrial Research Organisation) well-being diet, 5:2 diet, anti-inflammatory diet, liver-cleansing diet, gluten and dairy-free diet, and customised individual diet.

The use of common nutrition interventions, including recommendations for nutritional supplements, was further explored using binomial logistic regression. Recommendations for the use of nutritional supplements to patients amongst the participants were more likely amongst chiropractors who had some nutrition education in their undergraduate course, who were older, female and were familiar with the Australian Dietary Guidelines. Details are provided in Table 4.

**Table 4:** Multivariate model of factors associated with recommending nutritional supplements

Variable (reference)	Odds Ratio	Lower Confidence Interval	Upper Confidence Interval	*p*-Value
Australian Dietary Guidelines^a^ (knowledge)	2.531	1.007	6.362	0.048
Nutrition Education (yes)	3.774	1.003	14.201	0.049
Age (older)	4.410	1.429	13.614	0.010
Gender (male)	3.361	1.123	10.060	0.030

Variables Not significant: ‘Recommend Australian Guide to Healthy Eating’, ‘Nutrition Recommendations made’, ‘practice location (urban rural)’

^a^Practitioner familiar with the Australian Dietary Guidelines

Most of the chiropractors surveyed worked with other health professionals to provide nutritional guidance for their patients. Participants’ referrals to other health professionals included to general medical practitioners (94%), dietitians (93%), nutritionists (91%) and naturopaths (91%).

## Discussion

The response rate for this survey compares favourably with other similar studies of chiropractors in the US [24] and UK [25] and the sample was comparable to the demographic characteristics of the Australian chiropractic workforce [40]. Most chiropractors in this survey reported providing some nutritional advice as a part of their day to day practice. The study by Adams et al. found that nutrition was the second most popular topic ‘often’ discussed (51% of the chiropractors) as part of patient clinical care after discussions about physical activity (85%) [40]. In comparison, nutritional and dietary recommendations are frequently provided by 80–99% of chiropractors in the US [24] and 74% in the UK [25].

Nutrition is included as a core competency in the evidence-based preventive clinical care services in chiropractic education in the USA [41]. However a study of chiropractors in New York identified a need for a standardised national nutrition counselling protocol in the US to actively encourage chiropractors to apply nutrition in consideration of patients’ wellness [24]. The study by Adams et al. and the results of our study suggest that there would be interest in a in a similar resource in Australia to clarify the role of nutrition in chiropractic clinical care within Australia as to promote health and to prevent chronic disease [40].

A high percentage of chiropractic patients attend for neuro-musculoskeletal disorders including back and neck pain [24, 34, 40, 42] and this is reflected by the most frequently encountered health problem, inflammatory conditions in this study.

For condition-specific nutritional guidance, our study revealed significant variations between chiropractors with some providing nutritional advice specific to health conditions listed in the survey and others indicating they would ‘rarely’ or ‘never’ provide dietary advice for these conditions. These differences may be attributed to variation in education, knowledge, confidence and personal beliefs about the applicability of nutritional advice within the scope of chiropractic. It is possible that those who chose ‘never’ hold the opinion that nutritional advice is not within the scope of chiropractic care. This indicates a need for communication and consensus within the chiropractic educational institutes and profession to establish a clear framework for how nutritional guidance fits within chiropractic patient management.

The results of this study show that there is considerable interest in nutrition by chiropractors and because of the increasing importance of chronic disease in Australia it would be useful to establish a nutrition competency framework for chiropractic to provide evidence-based nutrition information and resources to their patients. This has been done by other health professions. Results from a survey of Australian general practitioners indicated that there was a lack of consistency in the training and integration of nutritional education and skills across different universities, and that the standard of nutrition education was largely determined by individual course facilitators at each university [43]. Another study examined the need for the delivery of appropriate nutrition education within Australian university medical schools in order to enable future general practice registrars to effectively support patients in making healthy dietary choices [44]. Based on the results from these previous studies, the Nutrition Competency Framework (NCF) for medical graduates was developed by several medical schools in Australia in consultation with the Dietetic Association of Australia [45]. The NCF consists of knowledge and skills competencies, mapped to the Australian Medical Council Graduate Outcome Statements.

The participants in our study reported using a variety of nutritional assessment methods during the initial consultation. Nutritional assessment methods enquired about in the questionnaire were: anthropometric measurements (e.g. height, weight, waist circumference), laboratory assessments (e.g. blood and urinalysis), assessment of physical appearance, dietary assessment (e.g. diet history, food diary, food-frequency questionnaires), asking patient’s perception of their health and nutritional status, alcohol consumption, smoking status, use of medications, herbal medicines and nutritional supplements.

A detailed assessment of patient’s nutritional status includes a combination of anthropometric and biochemical measurements, physical examination (including history) and dietary assessment [46]. The use of anthropometric measurements, height and weight (including the Body Mass Index, BMI) and waist circumference, and dietary assessments were not commonly reported by chiropractors in this survey. They are currently used for essential screening and assessment of nutritional status and included in the clinical practice guidelines for healthcare clinicians to provide relevant lifestyle and promotion of healthy eating especially in overweight and obese patients in Australia [46, 47]. It would be useful for chiropractors to routinely record the BMI and waist circumference in the initial patient consultation to assess general health risks associated with being overweight or obese.

In the US, the use of a food diary is commonly recommended as a dietary assessment method, although the time-restrictions of undertaking this method have been acknowledged [24, 48]. Although a food diary was also used by some participants in this survey, Australian chiropractors apparently view referral to a qualified nutrition professional more favourably regarding dietary assessment, which may be a more efficient use of their time.

### Resources used to provide nutritional advice

Almost all chiropractors in this survey are familiar with the Australian Dietary Guidelines., The NHMRC clinical practice guidelines recommend that the current Dietary Guidelines and the Australian Guide to Healthy Eating should be used as the basis of advice on nutrition for adults [46]. This could usefully be reinforced in a future nutrition skills framework for chiropractors.

Surveys of chiropractors in the US of their nutrition education and counselling practices report a need for dietitians to become involved in the nutrition-related practices of chiropractors as sources for information and referral [29, 49]. In our study, 35% of chiropractors reported using resources provided dietitians in their practices, reinforcing the importance of the way nutrition is perceived as beneficial for the health of chiropractic patients in both countries.

### Nutritional recommendations and interventions

The most frequent nutritional advice provided by Australian chiropractors in this study was related to nutritional supplements, reflecting the patterns found in the US [24] and Canada [34, 50]. Most chiropractors (95%) surveyed in Alberta (Canada) endorsed health product sales, and most (89%) were engaged in sales including nutritional supplements (68%) and vitamins (52%). The authors of those studies encouraged chiropractors to be aware of the national code of ethics and conduct in endorsing health products [50].

Nutritional supplements are known to have an important role in correcting some deficiencies such as iron, the use of calcium supplements by women to reduce the prevalence of osteoporosis, and folate for the prevention of neural tube defects [51, 52], but generally it is preferable to get all nutrient requirements from a healthy diet. However, nutritional supplements should not be the first call in the promotion of a healthy diet. In this study, the rate of recommendation of general dietary supplements appears higher compared to the provision of healthy eating advice [53, 54]. The rate of recommendation of supplements was 92%, and 72% of the respondents reported “always” or “frequently” discussed “making healthy food and drink choices” with their clients who have nutritional issues. This suggests a need to promote existing evidence-based Dietary Guidelines and its food guide, the Australian Guide to Healthy Eating, within the chiropractic profession.

Specific dietary advice was given by 28% of our respondents and this is comparable to 26% in a survey conducted in the US [28]. In contrast to the US, only one respondent in our study specified dietary risk factors such as reducing saturated fat and low sodium intake which were common nutritional advice reported in the US study. A recent UK survey indicated most chiropractors (80%) considered ‘improved eating’ was a lifestyle issue that they felt responsible to discuss, and ‘poor diet’ is a behaviour to be monitored. Therefore nutrition advice and/or resources including setting goals are considered as a part of preventive and health promotional care offered to patients [25]. In each of these countries the need to address chronic diseases associated with unhealthy eating is a major health priority and chiropractors play and important role. In Australia the equivalent advice could be offered by chiropractors using the Australian Guide to Healthy Eating.

Referral to a dietitian or nutritionist by chiropractors was relatively uncommon in the US at 15% [24]. However, in this study around 90% had referred to dietitians for more detailed information on nutrition problems or dietary plans. Australian chiropractors in this survey acknowledge the importance of nutrition in their clinical practice, especially for patients with chronic disease. Almost all respondents had some nutrition component in their professional education.

### Study limitations and further research

There are several limitations that need to be considered when interpreting the results of this study. The response rate was satisfactory for this type of survey and the demographic characteristics were similar to the Australian chiropractic workforce, however the nature of the online survey method meant that the number of questions had to be limited. There may also be the potential for response bias in the self-reported nature of the survey.

## Conclusions

Most chiropractors in Australia report providing nutritional guidance as a part of their day to day practice; however, there are some discrepancies between their dietary advice and the methods used to assess nutritional status. Chiropractors are likely to refer patients with nutritional issues to other health professionals with expertise in nutrition, such as dietitians. There are opportunities and responsibilities for the chiropractic profession in Australia to promote overall health and enhance patient care by promoting healthy eating and lifestyle and more research is required to explore and assess the contribution of chiropractors to such wider public health and prevention advice and strategies. Development of a framework for nutrition competency or nutritional guidance for entry level chiropractors such as the NCF for medical graduates may be useful in future for chiropractors in Australia to enable them to be actively involved in prevention of chronic disease and promotion of health and wellbeing of their patients.

## Abbreviations

ACORN: Australian Chiropractic Research Network
ADG: Australian Dietary Guidelines
AGHE: Australian Guide to Healthy Eating
CSIRO: Commonwealth Scientific and Industrial Research Organisation
FODMAP: Fermentable Oligosaccharides, Disaccharides, Monosaccharides and Polyols
GI: Glycaemic Index
GP: General Practitioner
NCF: Nutrition Competency Framework
NHMRC: National Health and Medical Research Council
SPSS: Statistical Package for the Social Sciences
